# Supplementary material for: Habitual Dietary Fiber Intake, Fecal Microbiota, and Hemoglobin A1c Level in Chinese Patients with Type 2 Diabetes
Source: Nutrients. 2022 Feb 27;14(5):1003. doi: 10.3390/nu14051003 (PMC8912884; doi:10.3390/nu14051003)
Supplement: Supplementary file 1 [file nutrients-14-01003-s001.zip › nutrients-1607645-supplementary.pdf]

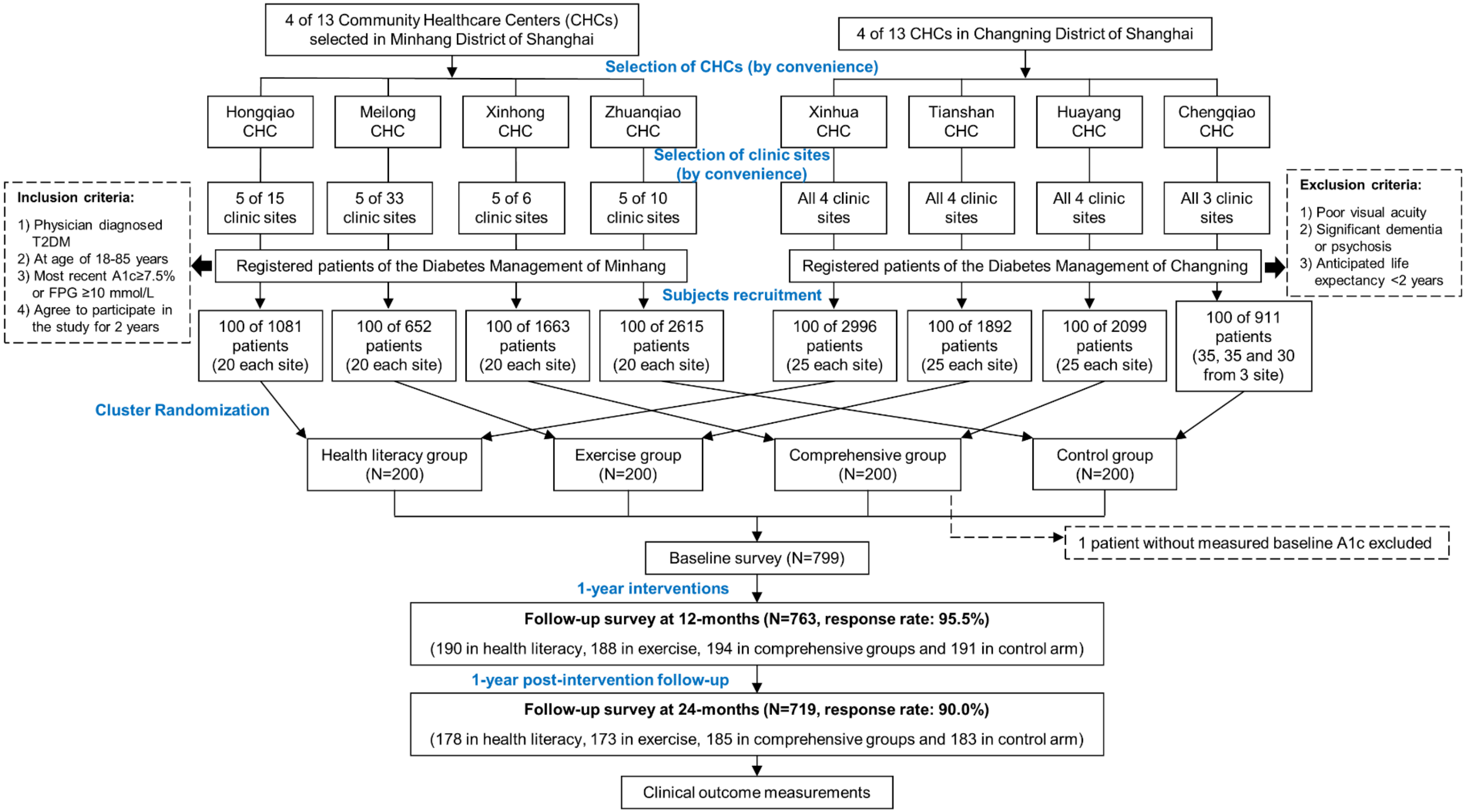

Supplementary Figure S1. Flow chart of the parent randomized controlled trial

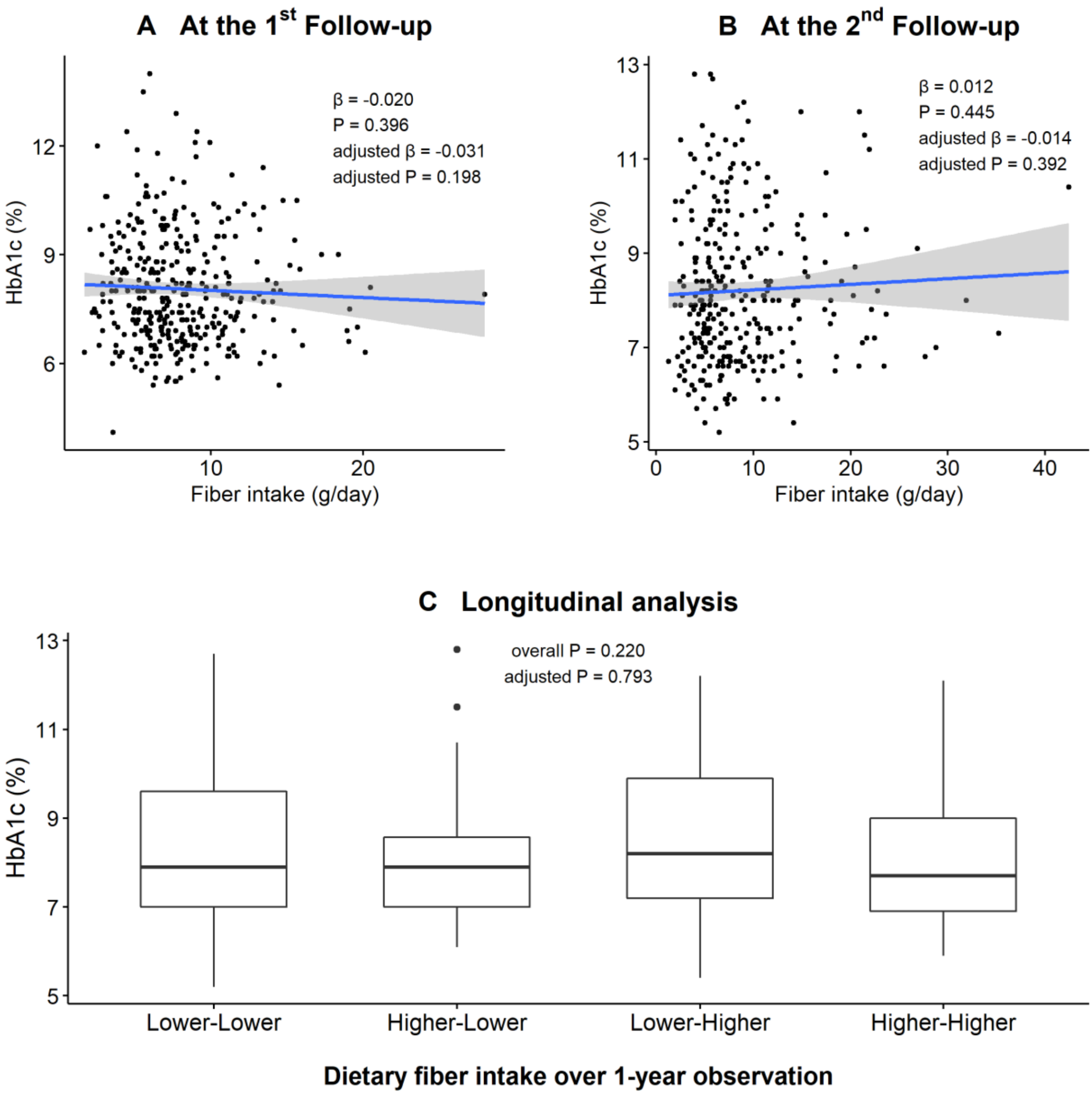

Supplementary Figure S2. Changes in HbA1c level along with dietary fiber intake within and across the first and the second follow-up surveys.

HbA1c level used in longitudinal analysis from the data in the second follow-up

General linear model adjusted for age, sex, educational level, duration of diabetes, intervention status in parent trial, physical activity level, energy intake, fat intake, and use of anti-diabetes agents within six months of the first follow-up (A), or the second follow-up (B), or over 1-year observation (C).

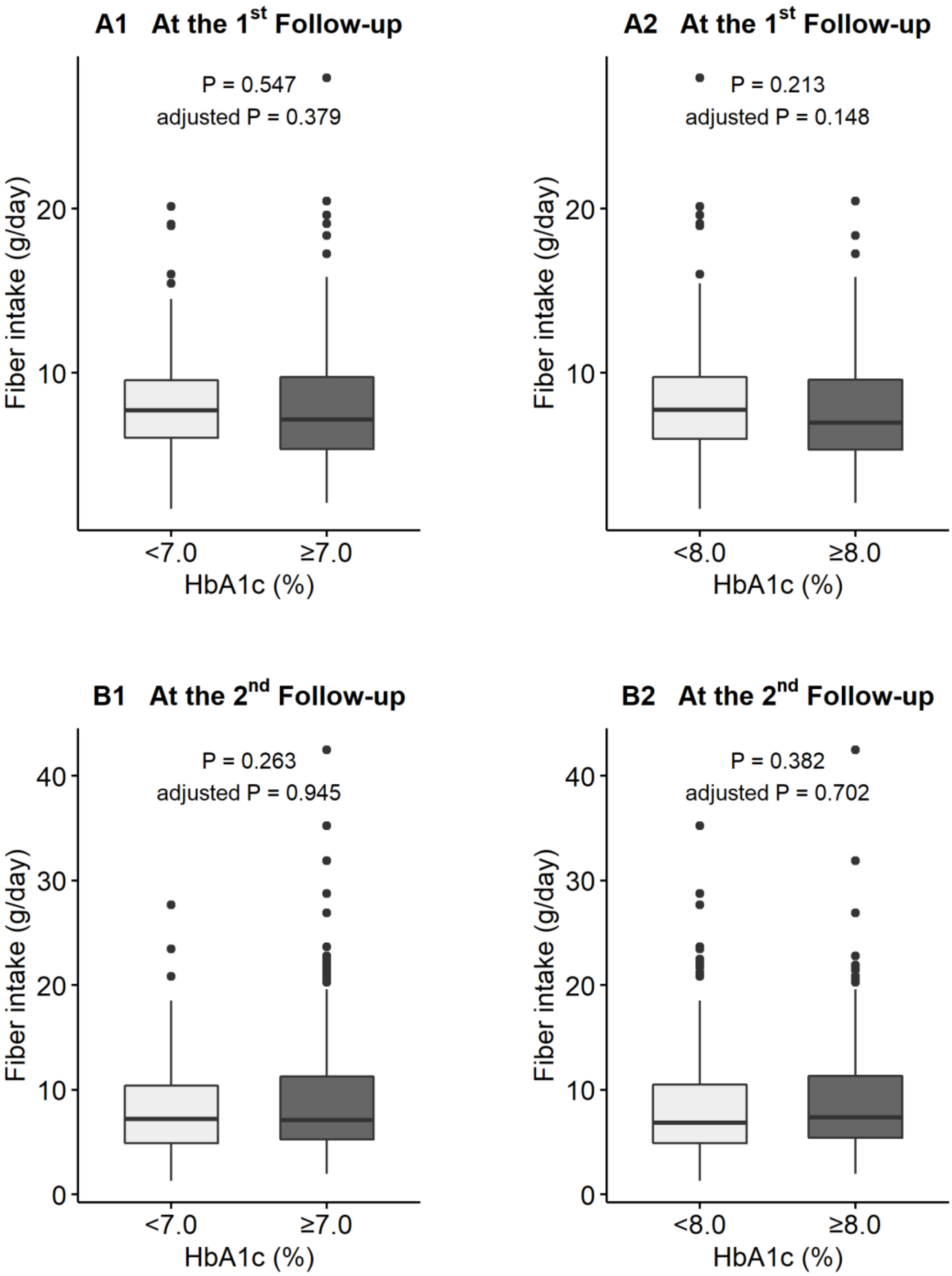

Supplementary Figure S3. Associations of dietary fiber intake with glycemic control status at the first and the second follow-up surveys.

General linear model adjusted for age, sex, educational level, duration of diabetes, intervention status in parent trial, physical activity level, energy intake, fat intake, and use of anti-diabetes agents within six months of the first follow-up (A) or the second follow-up (B).

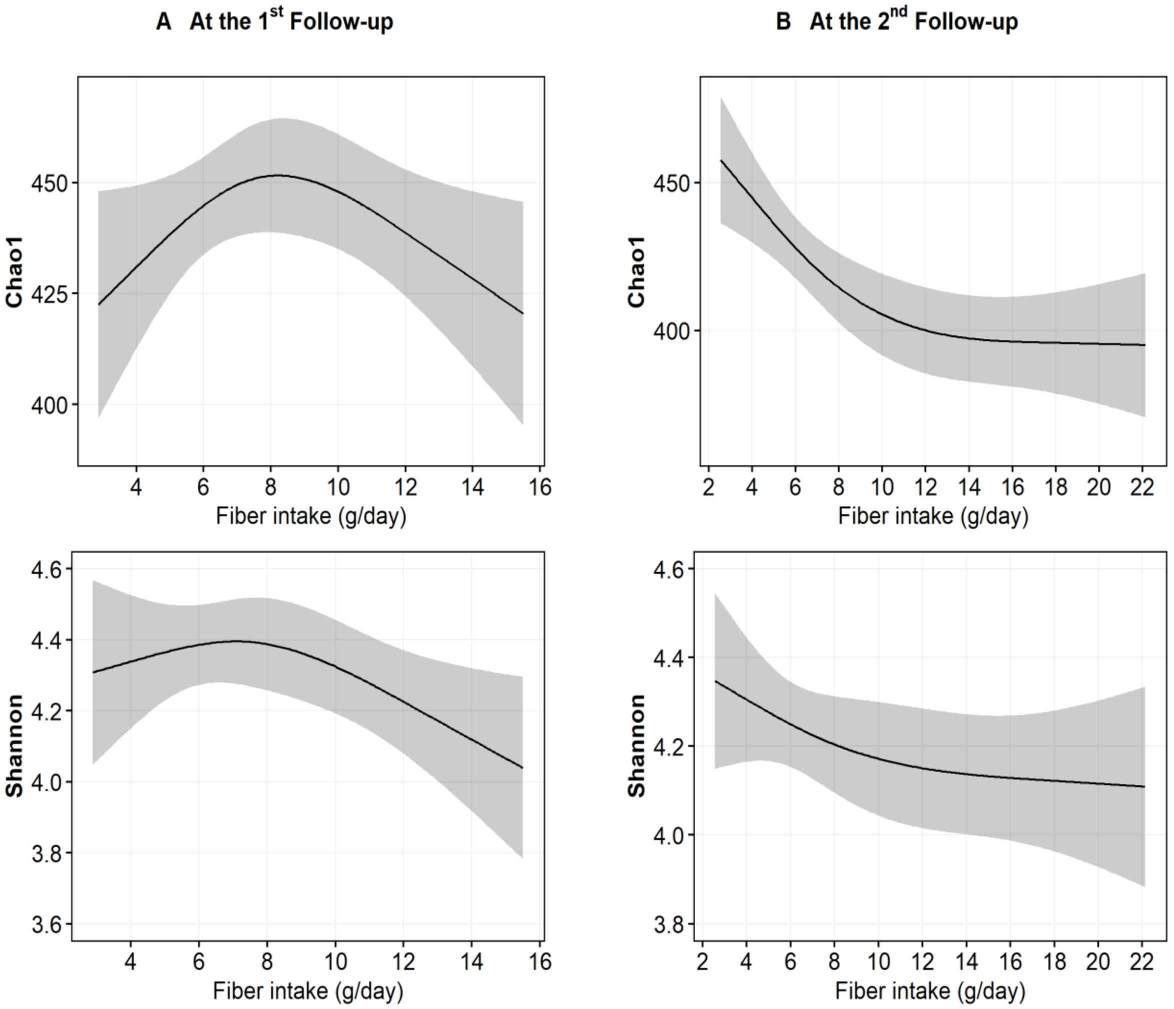

Supplementary Figure S4. Restricted cubic spline (RCS) curve of α-diversity of gut microbiota along with dietary fiber intake at a continuous scale at the first (A) and the second (B) follow-up.

Solid black lines presented predicted values of α-diversity, while grey shadow for 95% confidence intervals derived from RCS regressions with three knots.

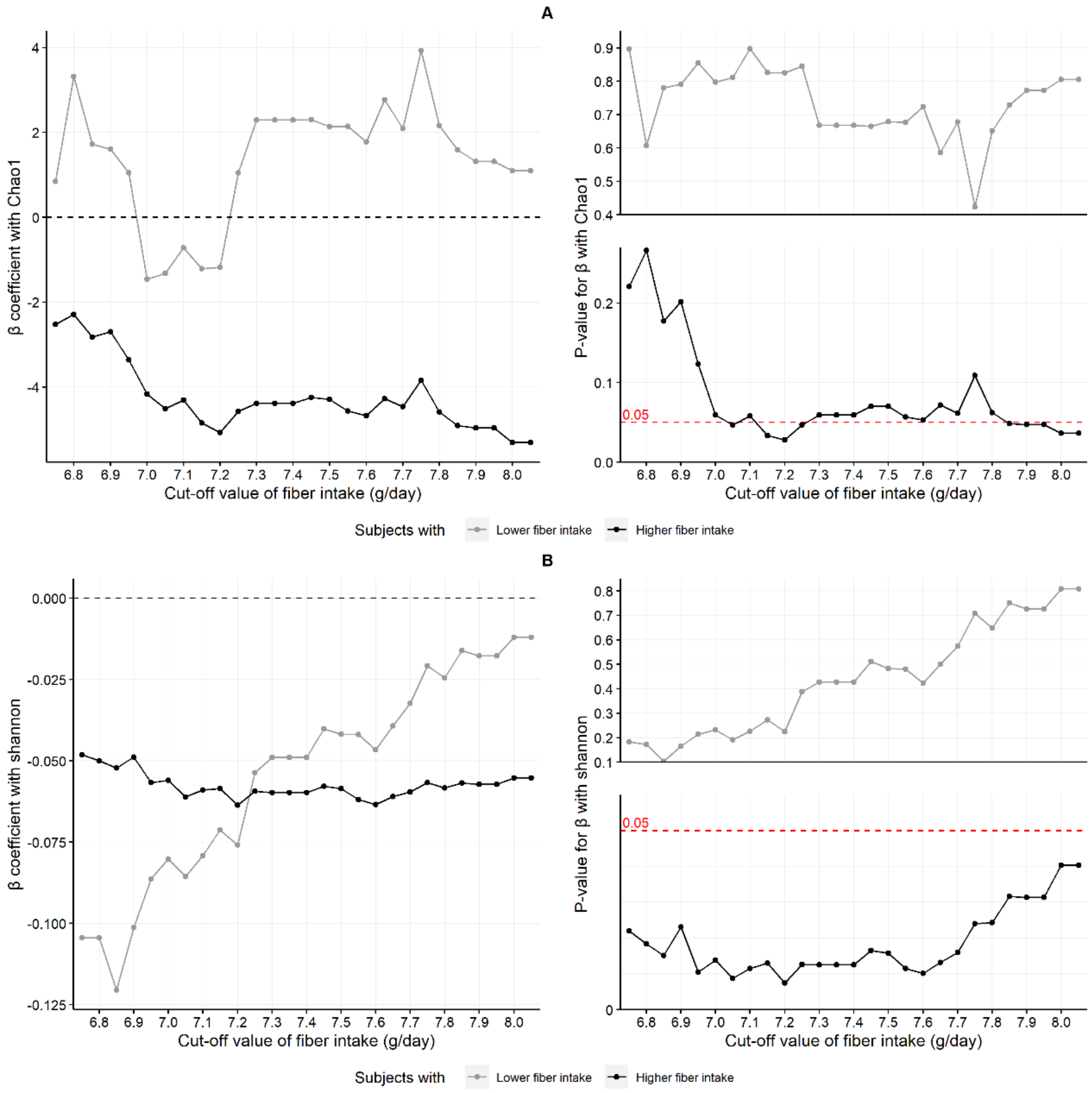

Supplementary Figure S5. Piecewise regression β coefficients of Chao1 (A) and Shannon (B) for the higher versus the lower dietary fiber intake at the first follow-up.

Lower or higher fiber intake defined as less than or no less than corresponding cut-off values of dietary fiber intake.

A At the 1st Follow-up

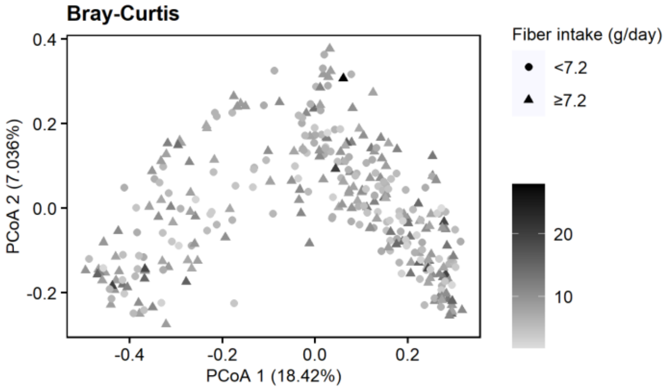

At the 1st Follow-up	PERMANOVA P-value
All subjects (n=325)	0.122
**By fiber intake (g/day)**	
Lower (< 7.2, n=153)	0.235
Higher (≥ 7.2, n=172)	0.150
Higher versus Lower	0.110

B At the 2nd Follow-up

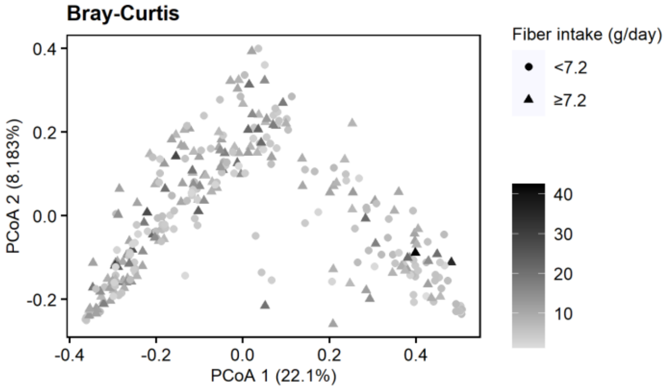

At the 2nd Follow-up	PERMANOVA P-value
All subjects (n=301)	0.295
**By fiber intake (g/day)**	
Lower (< 7.2, n=152)	0.524
Higher (≥ 7.2, n=149)	0.857
Higher versus Lower	0.014

C Longitudinal analysis

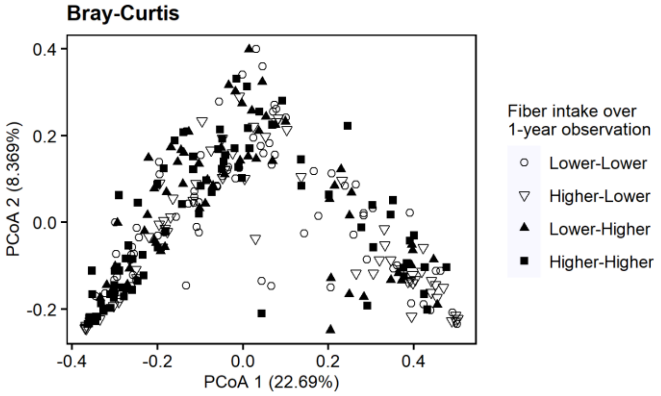

Longitudinal analysis	PERMANOVA P-value
Higher-Higher (n=82)	
versus	0.186
Lower-Lower (n=80)	

Supplementary Figure S6. Associations of dietary fiber intake with β-diversity of gut microbiota in Chinese diabetes patients.

β-diversity of gut microbiota in longitudinal analysis derived from data at the second follow-up.

P-values for Permutational Multivariate Analysis of Variance (PERMANOVA) adjusted for age, sex, education level, duration of diabetes, RCT group, physical activity level, intervention status in parent trial, energy intake, fat intake, use of anti-diabetes agents and NSAIDs within six months before stool collection.

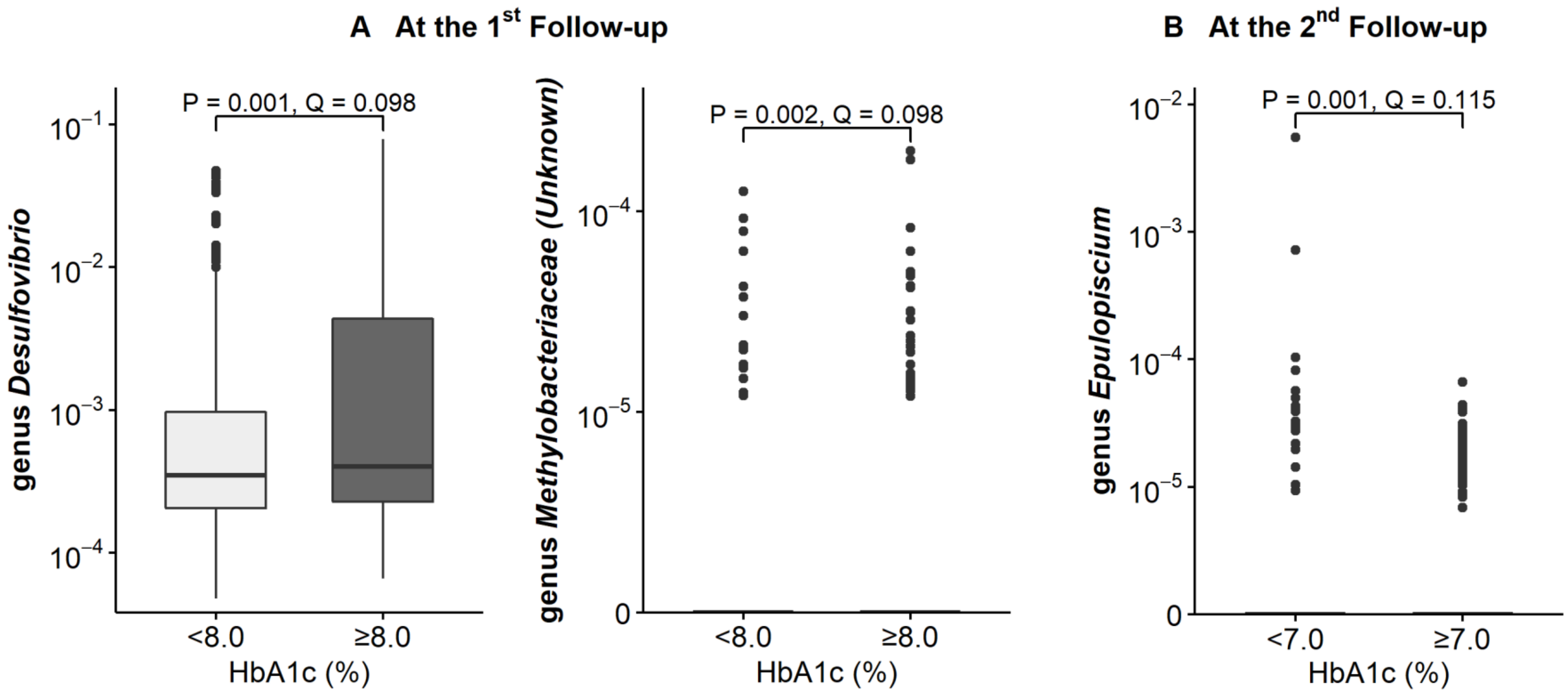

Supplementary Figure S7. Associations of relative abundances of taxa with glycemic status in Chinese diabetes patients.

Glycemic status classified by HbA1c level of 7.0% or 8.0%. Listed taxa with *Q*-values less than 0.15 only. All analyses in microbiome multivariable associations with linear models (MaAsLin) adjusted for age, sex, education level, duration of diabetes, intervention status in parent trial, physical activity level, energy intake, fat intake, use of anti-diabetes agents and NSAIDs within six months before stool collection.
